# Hyodeoxycholic acid inhibits colorectal cancer proliferation through the FXR/EREG/EGFR axis

**DOI:** 10.3389/fcell.2024.1480998

**Published:** 2025-01-06

**Authors:** Qiang Pang, Shansong Huang, Xiaodong Li, Jiaqing Cao

**Affiliations:** Department of Gastrointestinal Surgery, The Second Affiliated Hospital, Jiangxi Medical College, Nanchang University, Nanchang, China

**Keywords:** colorectal cancer, Hyodeoxycholic acid, FXR, EREG, EGFR

## Abstract

**Background:**

The high morbidity and mortality rates of colorectal cancer (CRC) have been a public health concern globally, and the search for additional therapeutic options is imminent. Hyodeoxycholic acid (HDCA) has been receiving attention in recent years and has demonstrated potent efficacy in several diseases. Nonetheless, the antitumor effects and molecular pathways of HDCA in CRC remain largely unexplored.

**Methods:**

In this study, we investigated how HDCA influences the growth potential of CRC cells using techniques such as flow cytometry, Edu assay, CCK-8, colony formation assay, Western blot analysis, and animal experiments.

**Results:**

It was found that HDCA treatment of CRC cells was able to significantly inhibit the proliferative capacity of the cells. Furthermore, it was discovered that HDCA primarily stimulated Farnesoid X Receptor (FXR) rather than Takeda G protein coupled receptor 5 (TGR5) to suppress CRC growth. It was also confirmed that HDCA inhibited the Epiregulin (EREG)/Epidermal Growth Factor Receptor (EGFR) pathway by activating FXR, and a negative correlation between FXR and EREG was analyzed in CRC tissue samples. Finally, *in vivo* animal studies confirmed that HDCA inhibited CRC proliferation without hepatotoxicity.

**Conclusion:**

Our findings indicate that HDCA suppresses the EREG/EGFR signaling route by activating FXR, thereby hindering the growth of CRC cells and demonstrating a tumor-inhibiting effect in CRC. This study may provide a new therapeutic strategy to improve the prognosis of CRC.

## 1 Introduction

Colorectal cancer (CRC) ranks among the prevalent gastrointestinal cancers globally, characterized by significant incidence and death rates (Sung et al., 2021). While existing treatments have somewhat enhanced the outlook for CRC patients, significant obstacles remain in developing effective CRC therapies. Consequently, it is crucial to identify essential elements and potential targets for CRC treatment, as well as drugs or natural substances that can accurately address them.

Lately, more researchers have concentrated on the connection between bile acids and cancer development (Cong et al., 2024; Zhu et al., 2023; Riscal et al., 2024). Cholesterol’s primary metabolites, bile acids, are produced in the liver and traveled to the intestine via the enterohepatic circulation. There, the intestinal microbiota partially transforms them into secondary bile acids. As an important signaling molecule in the organism, bile acids have been shown in many studies to play important roles in glucose homeostasis (Zheng et al., 2022; Huang et al., 2024), energy metabolism (Li et al., 2024), inflammation (Zhuang et al., 2024), and tumorigenesis and development (Riscal et al., 2024), in addition to their traditionally considered functions in lipid digestion. Hyodeoxycholic acid (HDCA) belongs to a type of secondary bile acid, which is found in high levels in pig bile and is the main active ingredient in the traditional Chinese medicine pig bile (Zhong et al., 2023). HDCA, present in modest amounts in humans and rodents, has recently been demonstrated to significantly reduce hyperlipidemia (Watanabe and Fujita, 2014), alleviate glucose metabolism (Zheng et al., 2021), and mitigate non-alcoholic fatty liver disease (Zhong et al., 2023). Nonetheless its role in CRC is unclear.

The Farnesoid X Receptor (FXR), similar to the Takeda G protein-coupled receptor 5 (TGR5), is primarily activated by bile acids and functions as a transcription factor encoded by the NR1H4 gene, predominantly found in the liver and intestines. To date, numerous studies have confirmed that bile acids exert biological functions by modulating the FXR. For instance, He et al. found that ursodeoxycholic acid alleviates acute lung injury by upregulating FXR expression, thereby inhibiting the p38 MAPK/NF-kB signaling pathway (He et al., 2024). Tschuck et al. demonstrated that bile acids activate FXR, leading to the suppression of lipid peroxidation and ferroptosis (Tschuck et al., 2023). Additionally, other research has reported that hyocholic acid increases the production and secretion of GLP-1 in enteroendocrine cells by simultaneously activating TGR5 and inhibiting FXR (Zheng et al., 2021). Numerous research papers have shown that FXR is crucial in the progression of CRC (Fu et al., 2019; Guo et al., 2022); Yu et al. showed that downregulation of FXR expression promoted CRC progression (Yu et al., 2022); in addition, another study found that FXR could play a tumor suppressor role in CRC by inhibiting Wnt/β-catenin signaling (Yu et al., 2020). Therefore, activation of FXR by bile acids may have a role in inhibiting CRC progression.

This study evaluated the effect of HDCA on the proliferative capacity of CRC cells *in vitro* and *in vivo*. And the relationship of HDCA mediating the Epiregulin (EREG)/Epidermal Growth Factor Receptor (EGFR) signaling pathway through FXR was explored.

## 2 Methods and materials

### 2.1 Patients and clinical specimens

Eight pairs of primary CRC and paracancerous tissue specimens were collected from the Second Affiliated Hospital of Nanchang University. All patients did not receive any treatment prior to their surgeries, and their tumors were in staged between II and IV. The collected specimens were used to detect the levels of EREG and FXR by Western blotting. The Ethics Committee of the Second Affiliated Hospital of Nanchang University granted approval for the research, and every participant gave their informed written consent.

### 2.2 Cell culture and transfection

Human CRC cell lines (HCT116 and DLD1) were purchased from China Fuheng Biotechnology Co. DLD1 was cultured in DMEM (Solarbio, China) medium at 37°C with 5% CO2, while HCT116 was cultured in 1640 medium.

CRC cells were inoculated in T25 culture flasks and transfected when the cell density was 60%–70%. Small interfering fragments (siRNA) and Lipofectamine 3,000 (Thermofisher, America) were firstly mixed with serum-free and penicillin-streptomycin-free medium, respectively, and subsequently the two were mixed and added to the culture flasks. Following a period of 6–8 h, the medium was substituted with one that included serum and penicillin-streptomycin for further incubation.

### 2.3 CCK-8 assay and lactate dehydrogenase (LDH) activity assay

The CRC cell line was mixed and resuspended with culture medium and inoculated in 96-well plates at 5000/100ul, while 100 µL complete medium without cells was set up (as a control). Following a 24-h period for cell adhesion to the wall, various doses of HDCA (Aladdin, China) were introduced. The optical density was measured at 0, 24, 48, and 72 h. Each well received 10 µL of CCK-8 reagent and was incubated for 2 h. Ultimately, the OD was read at 450 nm with an enzyme-linked immunosorbent assay reader.

CRC cells were inoculated in 96-well plates containing different concentrations of HDCA and cultured for 48 h. Then, the cells were incubated in PBS for 1 h. At the end of the incubation, LDH activity was determined according to the kit instructions.

### 2.4 5-ethynyl-2′-deoxyuridine assay

Again as described above first resuspend CRC cells in culture medium and inoculate in 96-well plates at 10,000 cells per 100 µL. The cells were given 8 h to adhere to the surface, followed by a 48-h treatment with the appropriate HDCA concentration. Cells were incubated with 5-ethynyl-2′-deoxyuridine (EdU, 10μM, UE, China) for 2 h, followed by fixation using 4% paraformaldehyde for 20 min. They were then permeabilized with 0.3% Triton X-100 for 30 min and thoroughly washed with BSA. Then, EdU staining was performed by incubating cells for 1 h, followed by DAPI staining for nuclear visualization. Ultimately, images were captured with a fluorescence microscope, and the count of Edu-positive cells was assessed through ImageJ software.

### 2.5 Colony formation assay

For the colony formation assay, 1000 CRC cells were seeded into 6-well plates and grown for approximately 2 weeks in a full medium with a modified HDCA concentration. Once the colonies could be seen without a microscope, they were preserved using 4% paraformaldehyde and then dyed with 0.1% crystal violet. Finally, the colonies were photographed and analyzed using ImageJ software.

### 2.6 Flow cytometry assay

A cell cycle analysis was conducted utilizing the Cell Cycle Assay Kit from UE, China. The treated cells were digested and resuspended and fixed using 70% ethanol for more than 2 h (placed at 4°). Once fixation was done, the cells were rinsed with PBS, 500 µL of the working solution was introduced, and the cells were left to incubate at room temperature, shielded from light for 30–60 min, before being analyzed using flow cytometry.

### 2.7 Western blotting

Proteins were isolated with RIPA buffer supplemented with protease and phosphatase inhibitors, and their concentrations were determined using a BCA assay kit from Beyotime, China. Proteins were isolated using 10% SDS-PAGE and then moved to PVDF membranes, with transfer duration adjusted based on the target molecular weight. Membranes were blocked with 5% BSA for 1 hour at room temperature, followed by an overnight incubation with the primary antibody at 4°C. The next day, the secondary antibody was combined with the primary antibody for 1 hour at room temperature, followed by development and photography. The primary antibodies utilized in the experiments were anti-NR1H4 (1:2000, Proteintech, China); anti-TGR5 (1:500, Proteintech, China); anti-GAPDH (1:1000, Servicebio, China); anti-cyclin D1 (1:5000, Proteintech, China); anti-CDK6 (1:5000, Proteintech, China); anti-EREG (1:1000, CST, USA); anti-AREG (1:500, Proteintech, China); anti-Tublin (1:1000, Proteintech, China); anti-EGFR (1:1000, CST, USA); and anti-Phospho-EGFR (1:1000, CST, USA).The secondary antibody was sheep anti-rabbit (or mouse) Ig G (1:8000, UE, China).

### 2.8 qPCR

qPCR was performed to assess mRNA expression levels, following established protocols detailed in prior studies (Pang et al., 2024). In summary, RNA was initially isolated from cells with Trizol reagent, and then cDNA was produced through reverse transcription. In the end, qPCR (quantitative real-time PCR) was conducted. The mRNA levels were evaluated using the 2^−ΔΔCT^ technique, with GAPDH serving as the control. The primer sequences were as follows: FXR forward 5′-ACT​TCC​GTC​TGG​GCA​TTC​TGA​C-3′ and reverse 5′-GCT​GTA​AGC​AGA​GCA​TAC​TCC​TC-3′; AREG forward 5′-GCA​CCT​GGA​AGC​AGT​AAC​ATG​C-3′ and reverse 5′-GGC​AGC​TAT​GGC​TGC​TGC​TAA​TGC​A-3′; EREG forward 5′-CTT​ATC​ACA​GTC​GTC​GGT​TCC​AC-3′ and reverse 5′-GCC​ATT​CAG​ACT​TGC​GGC​AAC​T-3′.

### 2.9 *In vivo* xenograft experiments

HCT116 cells were resuspended in 100 μL of medium (5 × 10^6^ cells/100 µL) and administered subcutaneously into male BALB/c nude mice aged 4–5 weeks. A week post-injection, the mice were divided into three groups: control, HDCA-75 mg/kg, and HDCA-150 mg/kg, with three mice in each group. Mice received gavage injections on alternate days for 15 days. Tumor size was assessed every 4 days and computed using the formula: volume (mm^3^) = length × width^2^ × 0.52. Mice were necropsied, and the tumors were weighed, paraffin-embedded, and subjected to tissue staining. All animal studies were approved by the Animal Ethics Committee of Nanchang University (RYE2024061101).

### 2.10 Statistical analysis

GraphPad Prism 9 was utilized for conducting all statistical evaluations. ImageJ was used for characterization of the Edu assay and colony formation assay. The data were presented as the mean plus or minus the standard deviation and evaluated with a two-tailed Student’s t-test. A *p*-value below 0.05 was considered statistically significant.

## 3 Results

### 3.1 Effects of HDCA on HCT116 and DLD1 cell viability

**FIGURE 1:**
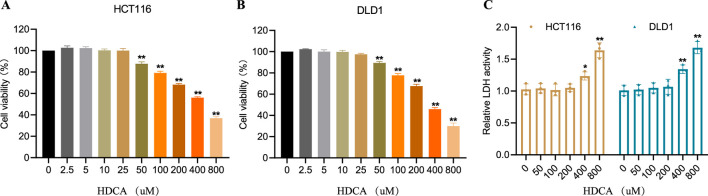
Selection of suitable concentrations of Hyodeoxycholic acid (HDCA). **(A, B)**, Cell viability of HDCA at different concentrations (0, 2.5, 5, 10, 25, 50, 100, 200, 400, and 800 μM) was determined by CCK8 assay for HCT116 and DLD1 after 48 h. **(C)**, Evaluation of HDCA cytotoxicity on HCT116 and DLD1 by measuring lactate dehydrogenase (LDH) activity. *Represents comparisons with concentration 0 μM; *p < 0.05, **p < 0.01.

To explore how HDCA impacts CRC cells, we initially conducted CCK-8 assays to assess the viability of HCT116 and DLD1 cells. The 2 cell lines were treated with appropriate concentrations of HDCA (0, 2.5, 5, 10, 25, 50, 100, 200, 400, and 800 μM) for 48 h in order to facilitate the selection of optimal treatment conditions. The results showed that HDCA exhibited an inhibitory effect on CRC cells when the concentration of HDCA started from 50 μM (Figures 1A, B). In addition, our lactate dehydrogenase assay revealed significant toxicity to CRC cells at concentrations of 400 and 800 μM (Figure 1C). Therefore, we finally chose 100 and 200 μM as the appropriate concentrations for subsequent studies.

### 3.2 HDCA inhibits proliferation of CRC cells

**FIGURE 2:**
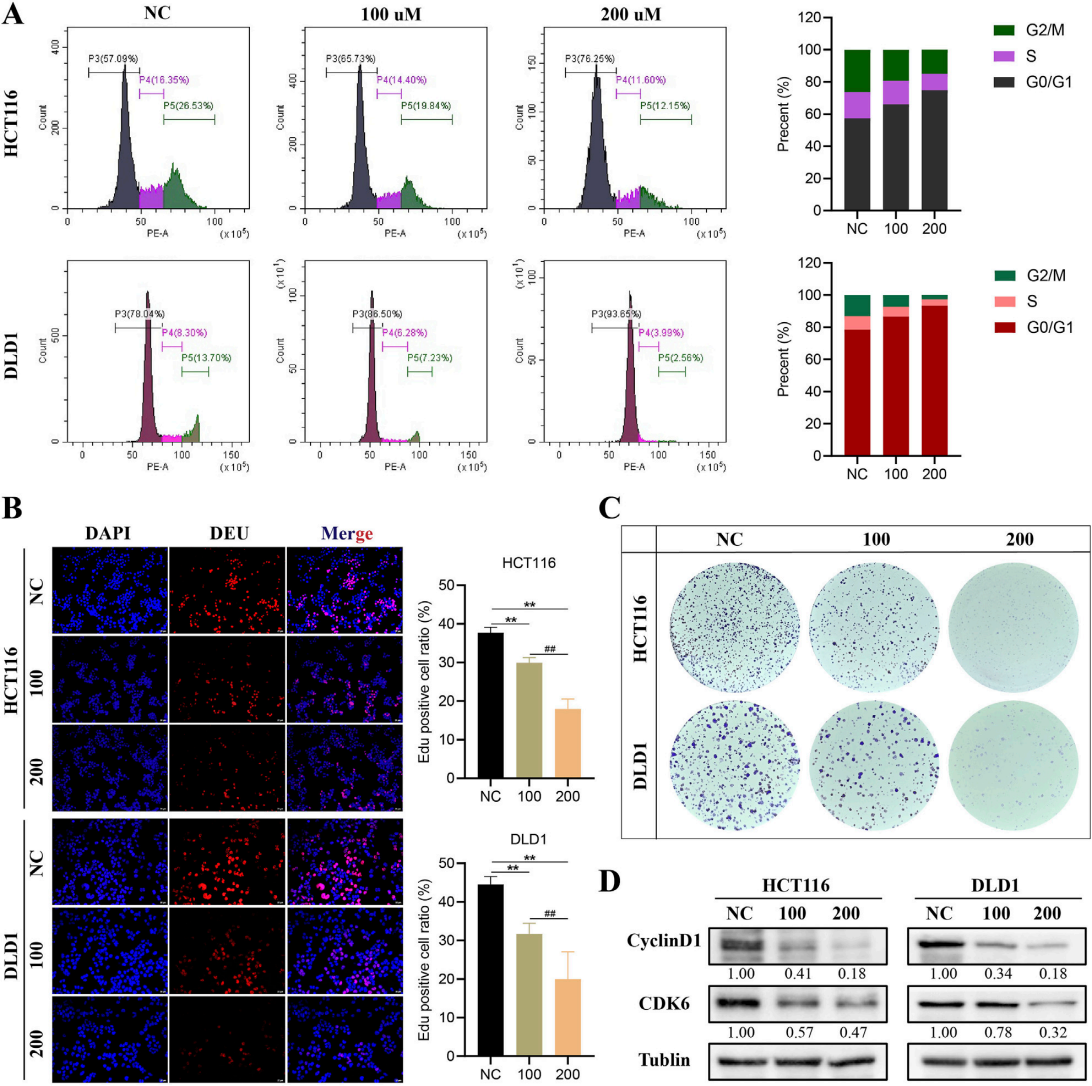
HDCA inhibits the proliferation of colorectal cancer (CRC). **(A)**, Flow cytometry assay for cell cycle analysis of CRC cells treated with different HDCA concentrations (100 and 200 μM) for 48 h. **(B)**, Edu assay to detect the effect of HDCA on the proliferative capacity of CRC cells. **(C)**, clonogenic generation of HCT116 and DLD1 cells cultured with the indicated HDCA concentrations. Assay. **(D)**, Western blotting assay of HDCA-treated CRC cells for 48 h on cylinD1 and CDK6 expression. *Represents comparisons with NC; # represents comparisons between 100 and 200 μM; *p < 0.05, **p < 0.01, ##*p* < 0.01.

To clarify the impact of HDCA on the growth of CRC cells, we conducted flow cytometry analysis, revealing that HDCA induced CRC cells to halt at the G0/G1 phase (Figure 2A). In addition, the proliferative ability of CRC cells was also confirmed to be significantly diminished in a concentration-dependent manner after the addition of HDCA by Edu and flow cytometry assay (Figures 2B, C). At the same time, the levels of cyclinD1 and CDK6, proteins associated with the cell cycle, were observed to decline as HDCA concentration rose (Figure 2D). In summary, HDCA can inhibit the proliferation ability of CRC cells.

### 3.3 HDCA activates FXR but not TGR5 to exert tumor suppressor effects

**FIGURE 3:**
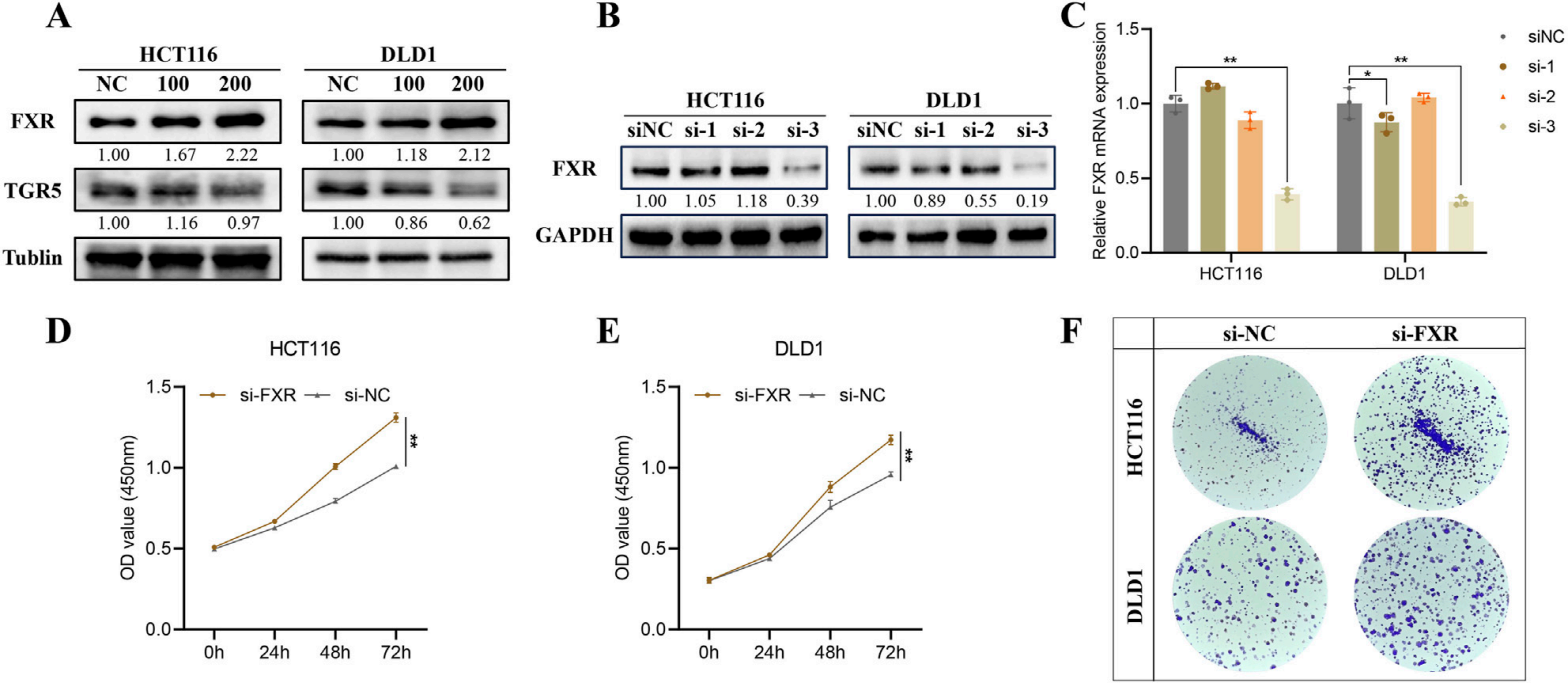
HDCA activates Farnesoid X Receptor (FXR) but not the Takeda G protein-coupled receptor 5 (TGR5). **(A)**, The expression of FXR and TGR5 was detected after the appropriate concentration of HDCA acted on CRC cells for 48 h. **(B, C)**, Western blotting and qPCR screening of siFXRs knocking down the FXR. **(D, E)**, CCK-8 assay to detect the proliferative capacity of the cells after knocking down of the FXR by HCT116 and DLD1, respectively. **(F)**, Colony formation assay after HCT116 and DLD1 knockdown of FXR. *p < 0.05, **p < 0.01.

We examined the two most common receptors for bile acids, FXR and TGR5, and found that HDCA mainly activated the expression of FXR, while the activation of TGR5 was not obvious (Figure 3A). In addition, we analyzed the oncogenicity of FXR in CRC, firstly we screened for effective knockdown of siRNAs of FXR by Western blotting and qPCR, and the results indicated that siFXR-3 significantly knocked down the expression of FXR (Figures 3B, C). Later, we verified through CCK-8 and colony formation assays that reducing FXR levels could accelerate CRC development (Figures 3D–F).

**FIGURE 4:**
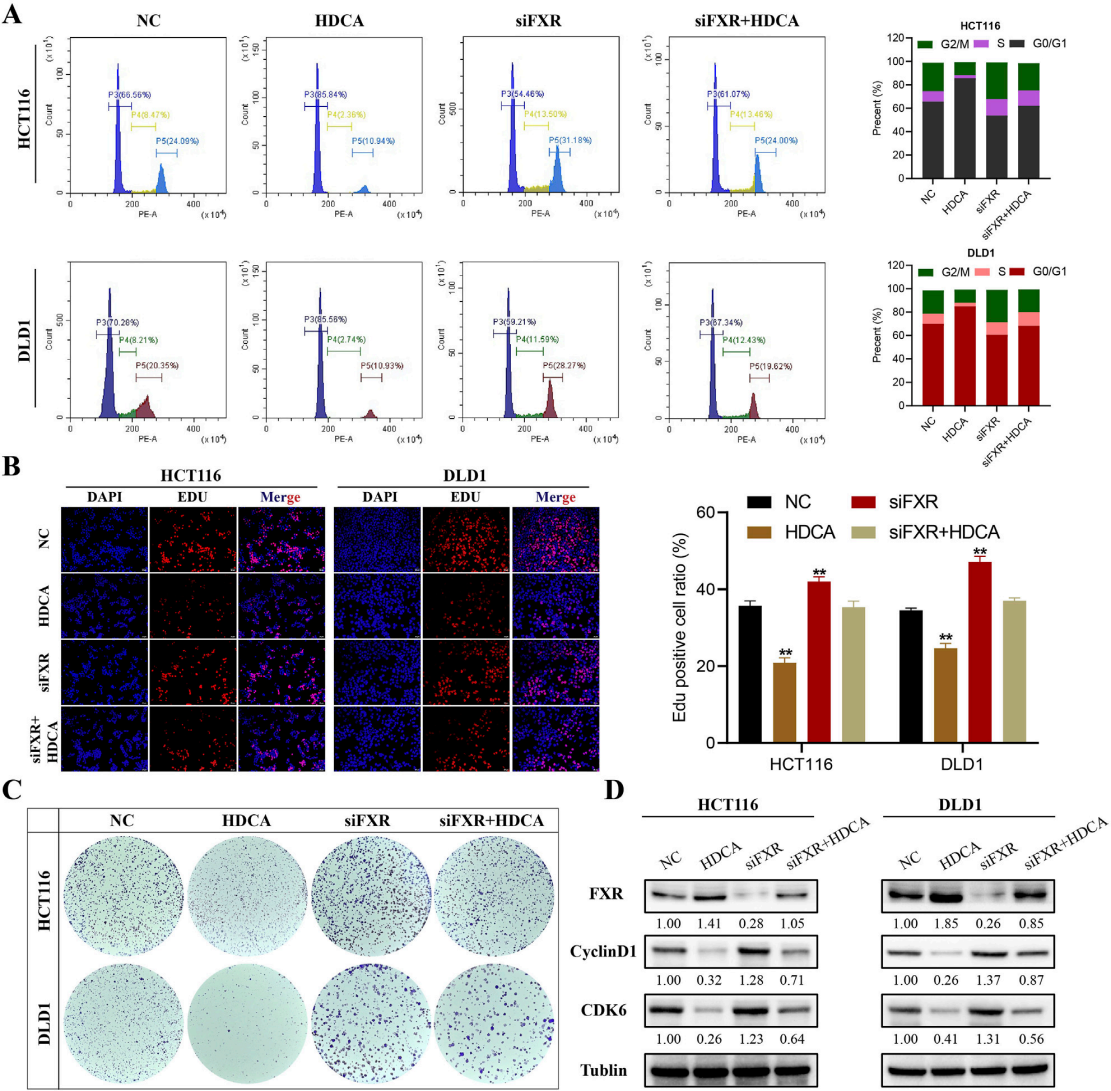
HDCA inhibition of CRC proliferation is dependent on FXR. **(A)**, Detection and analysis of cell cycle after 200 μM HDCA and/or siFXR treatment; **(B)**, Edu assay to detect the effect of 200 μM HDCA and/or siFXR treatment on the proliferative capacity of CRC cells; **(C)**, Colony formation assay of 200 μM HDCA and/or siFXR-treated cultured HCT116 and DLD1 cells. **(D)**, Western blotting analysis of FXR, Cyclin D1, and CDK6 in CRC cells after 200uM HDCA and/or siFXR treatment. *Represents comparison with NC; *p < 0.05, **p < 0.01.

To further elucidate the exact role of FXR in the inhibition of CRC proliferation by HDCA, we gave HDCA stimulation treatment while knocking down FXR. The flow cytometry analysis demonstrated that HDCA activation counteracted the proliferative influence of siFXR on CRC cells, causing a shift in the cell cycle from the G0/G1 phase to the G2/M phase (Figure 4A). At the same time, findings from the Edu and colony formation tests indicated that HDCA could counteract the impact of siFXR on the growth potential of CRC cells (Figures 4B, C). In addition, we also detected the expression of cycle-related proteins cyclinD1 and CDK6, and the results were consistent with the above (Figure 4D). Taken together, all these findings can indicate that HKDCA activates FXR but not TGR5 to inhibit CRC cell proliferation.

### 3.4 HDCA activates the FXR/EREG/EGFR pathway

**FIGURE 5:**
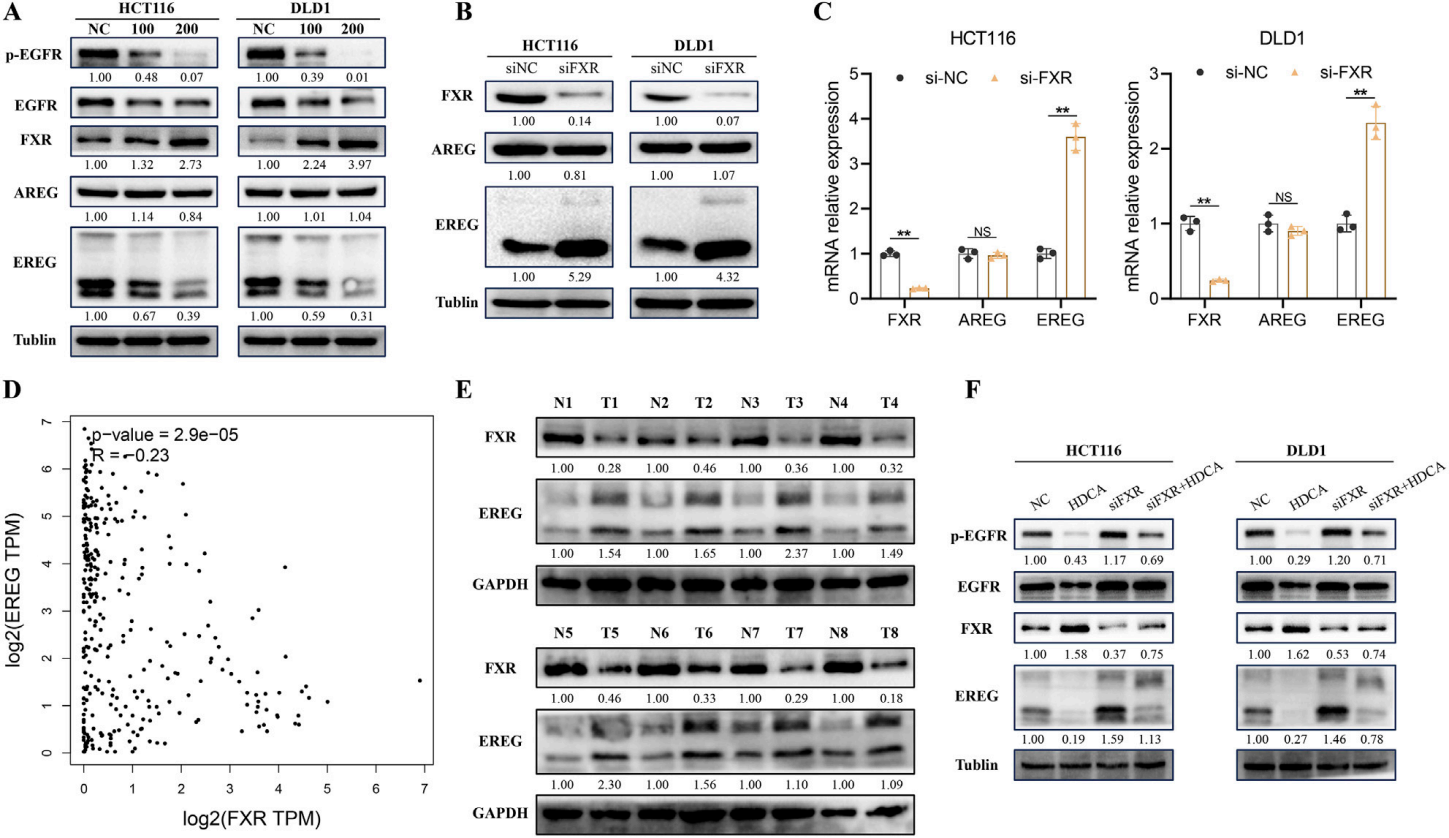
HDCA inhibits the Epiregulin (EREG)/Epidermal Growth Factor Receptor (EGFR) pathway. **(A)**, Western blotting analysis of FXR, AREG, EREG, EGFR, and p-EGFR (phosphorylated EGFR) after 48 h of action of the indicated concentrations of HDCA on CRC cells; **(B)**, Western blotting analysis of FXR, AREG, and EREG in CRC cells after knockdown of FXR; **(C)**, mRNA analysis of FXR, AREG, and EREG in CRC cells after knockdown of FXR. **(D)**, GEPIA2 analysis of the correlation between EREG and FXR; **(E)**, Western blotting of FXR and EREG in eight pairs of CRC tissue samples. **(F)**, Western blotting analysis of FXR, EREG, EGFR and p-EGFR in CRC cells after 200 uM HDCA and/or siFXR treatment Blot analysis. *p < 0.05, **p < 0.01.

Earlier research indicated a strong link between EGFR and tumor cell growth, prompting us to investigate EGFR and its phosphorylated form in CRC cells treated with HDCA. Our findings revealed that HDCA suppressed EGFR phosphorylation (Figure 5A). In addition, we detected two common ligands of EGFR, Amphiregulin (AREG) and EREG, and found that HDCA significantly decreased the expression of EREG, while it did not have a significant effect on AREG (Figure 5A). Consequently, we proposed that HDCA suppresses the growth of CRC cells by downregulating EREG and thereby preventing EGFR activation. Considering that bile acids can activate the transcriptional activity of FXR after the action of FXR, we hypothesized that FXR may inhibit the transcription of EREG and then play the role of cancer inhibition. Consequently, we examined the alterations in EREG and AREG using qPCR and Western blotting following FXR knockdown. The findings indicated a notable rise in both protein and mRNA levels of EREG post-FXR knockdown, whereas AREG levels remained largely unchanged (Figures 5B, C). Analysis by GEPIA2 website also revealed that FXR showed a significant negative correlation with EREG (Figure 5D). Furthermore, to enhance our validation, we analyzed eight pairs of CRC tissue samples and discovered that FXR levels were notably reduced in tumor tissues compared to adjacent non-cancerous tissues, whereas EREG levels were higher in the tumor tissues (Figure 5E). Therefore, we hypothesized that FXR might be a transcriptional repressor of EREG. In conclusion, to confirm that HDCA-induced activation of the EGFR pathway relied on FXR, we conducted Western blot analyses. The results indicated that FXR knockdown alone enhanced EGFR phosphorylation, but when FXR knockdown was combined with HDCA treatment, EGFR phosphorylation was partially suppressed (Figure 5F). Therefore, we conclude that HDCA can inhibit EGFR phosphorylation through the FXR receptor and thus inhibit the expression of EREG.

### 3.5 HDCA inhibits CRC proliferation *in vivo*

**FIGURE 6:**
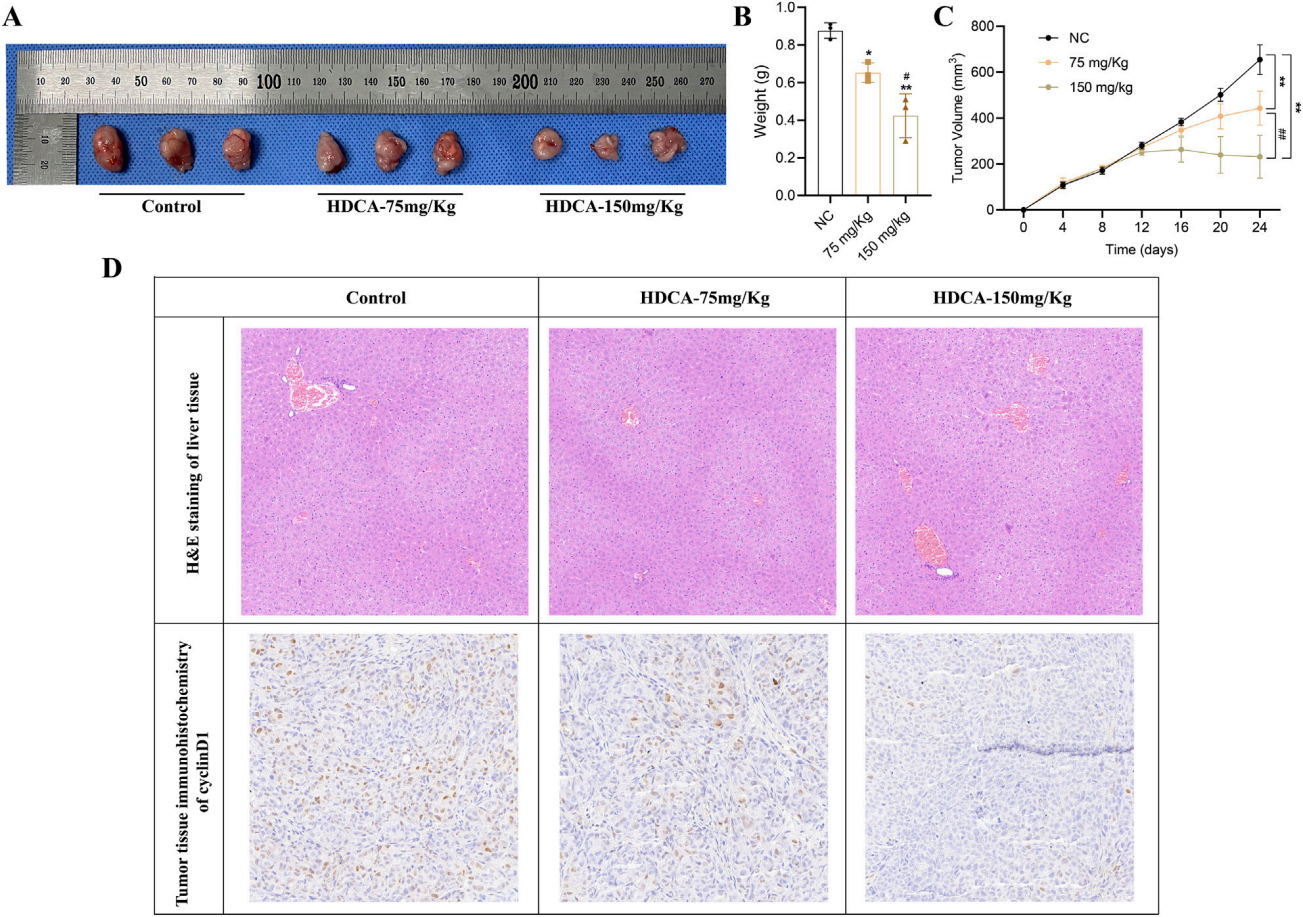
HDCA inhibits CRC proliferation *in vivo*. **(A)**, Tumor size of nude mice after administration of different concentrations (75 mg/kg and 150 mg/kg) of HDCA. **(B)**, weight analysis of the tumors. **(C)**, volumetric analysis of the tumors. **(D)**, HE staining and immunohistochemistry analysis of the tumor tissues. *Represents comparison with NC; # represents comparison between 75 mg/kg and 150 mg/kg; *p < 0.05, **p < 0.01, #*p* < 0.05, ##*p* < 0.01.

To further confirm the inhibitory effect of HDCA on CRC proliferation, we verified it by *in vivo* animal experiments. First, we inoculated CRC cells (HCT116) under the skin of nude mice, and when the subcutaneous tumors of nude mice were fully formed on the eighth day, HDCA was administered to the nude mice for 15 consecutive days. Research indicated a notable decrease in both tumor size and mass in nude mice, correlating with the dosage of HDCA administered (Figures 6A–C). In addition, in order to detect whether HDCA has liver damage, we performed HE staining on the livers of nude mice and found that there was no obvious liver function damage (Figure 6D). At the same time, immunohistochemistry was used to measure cyclinD1 levels in tumor samples, revealing that cyclinD1 expression diminished as HDCA concentration rose (Figure 6D). Thus, we determined that HDCA can safely prevent the growth of CRC cells.

## 4 Discussion

This research demonstrated that HDCA could suppress the growth of CRC cells and lack of liver toxicity through animal testing. In addition, it was found that HDCA could inhibit the EREG/EGFR signaling pathway by activating the expression of FXR, and then inhibit the proliferation of CRC.

An expanding body of research has verified the involvement of bile acids in cancer formation. However, the role of bile acids in tumor progression varies, and some bile acids may have a role in promoting tumor progression, while others have efficacy in inhibiting tumor progression. In one study, Taurodeoxycholic acid was found to promote malignant progression of gallbladder cancer through activation of YAP1 (Yang et al., 2023). Zhu et al. showed that deoxycholic acid could promote proliferation and invasiveness of CRC cells (Zhu et al., 2012).On the other hand, a study by zhang et al. demonstrated that ursodeoxycholic acid could inhibit the malignant progression of CRC through the TGR5-YAP axis (Zhang et al., 2021). However, some bile acids may play completely opposite roles in different tumors, such as lithocholic acid. In CRC, lithocholic acid promotes malignant progression and metastasis as a tumor promoter (Nguyen et al., 2017). However, Li et al. research indicated that lithocholic acid can suppress gallbladder cancer growth by disrupting glutamine metabolism (Li W. et al., 2022), and furthermore, it has demonstrated therapeutic benefits in treating breast cancer (Luu et al., 2018). In summary, the role of bile acids in tumor progression is complex and tissue-specific. Synthesized in the liver, bile acids bind to plasma proteins and enter the systemic circulation, distributing to various organs. The concentration and composition of bile acids differ across tissues, leading to diverse biological functions (Cai et al., 2022; Jia et al., 2024), which may account for their tissue-specific effects. Moreover, the concentration of bile acids is crucial in maintaining physiological homeostasis; appropriate levels stabilize bodily functions, while excessive or insufficient concentrations can disrupt this balance. This variability underscores the importance of investigating specific bile acids, such as HDCA, within particular contexts.

HDCA, a secondary bile acid, is highly abundant in porcine bile. Although HDCA is relatively less abundant in humans, several studies have confirmed its role in a variety of diseases. HDCA is effective in treating atherosclerosis and lowering cholesterol levels in mice (Sehayek et al., 2001). HDCA improves glucose homeostasis in diabetic mice via TGR5 and FXR receptors. Furthermore, multiple research papers have shown that HDCA can help reduce non-alcoholic fatty liver disease (Zhong et al., 2023; Kuang et al., 2023). These studies basically lead to the assumption that HDCA may be a beneficial bile acid, and thus our team hypothesized that HDCA may have a role in inhibiting tumor progression. Through *ex vivo* experiments, we verified that HDCA can suppress the growth of CRC cells and assessed its hepatotoxicity to ensure that HDCA effectively and safely inhibits CRC cell proliferation.

To investigate how HDCA suppresses CRC growth, we examined its impact on FXR and TGR5 receptors, ultimately determining that HDCA curbs CRC proliferation via FXR activation, not TGR5. FXR plays an important role in CRC, and it appears logical that HDCA-induced activation of FXR would have a tumor-inhibiting impact on CRC. In previous studies, bile acids have been shown to contribute to the EGFR pathway (Bhat et al., 2018; Raufman et al., 2008), and we evaluated the effect of HDCA on the EGFR pathway, and HDCA inhibited phosphorylation of EGFR. To further investigate the mechanisms by which HDCA affects the EGFR pathway, we evaluated its impact on the EGFR ligands EREG and AREG. Li et al. demonstrated that inhibiting EREG/EGFR expression exerts antitumor effects (Li M. et al., 2022). Similarly, Nakamura et al. found that downregulation of the EREG/EGFR/mTOR complex one signaling pathway suppresses gallbladder cancer progression (Nakamura et al., 2023). Additionally, Jiang et al. reported that AREG promotes lung cancer proliferation through the EGFR/PI3K/AKT/mTOR signaling pathway (Jiang et al., 2024). These studies collectively suggest that AREG and EREG, via EGFR, have crucial biological functions. Moreover, a Japanese study identified low-affinity EGFR ligands as signaling mediators in epithelial collective cell migration (Deguchi et al., 2024), further underscoring the importance of AREG and EREG as EGFR ligands. Further exploration of how HDCA inhibits EGFR phosphorylation revealed that FXR plays an important role, and the study confirmed that FXR may inhibit EREG expression through transcriptional suppression of EGFR phosphorylation. Unfortunately, however, we were not able to further explore the specific regulatory mechanism between FXR and EREG in depth, which deserves further investigation.

The present study still has some limitations. Firstly, this study failed to fully elucidate the relationship between FXR and EREG; secondly, the number of nude mice used in the *in vivo* experiments of this study was relatively small, and no *in vivo* animal experiments were performed in response. Finally, while our results indicate no observable liver toxicity at the tested doses of HDCA, these findings are preliminary. Comprehensive safety evaluations, including chronic toxicity studies, are necessary to establish HDCA as a therapeutic agent. Although there are some constraints, the experimental evidence presented in this research still shows that HDCA can prevent the growth of CRC.

To sum up, this research showed that HDCA can suppress the EREG/EGFR signaling route by activating FXR, thereby hindering the growth of CRC. Therefore, HDCA could be an alternative as a potential therapeutic intervention for CRC.

## Data Availability

The original contributions presented in the study are included in the article/supplementary material, further inquiries can be directed to the corresponding author.
